# Carbohydrate Loading Followed by High Carbohydrate Intake During Prolonged Physical Exercise and Its Impact on Glucose Control in Individuals With Diabetes Type 1—An Exploratory Study

**DOI:** 10.3389/fendo.2019.00571

**Published:** 2019-08-21

**Authors:** Stig Mattsson, Johan Jendle, Peter Adolfsson

**Affiliations:** ^1^Institute of Medical Sciences, Örebro University, Örebro, Sweden; ^2^Department of Pediatrics, The Hospital of Halland, Kungsbacka, Sweden; ^3^Institute of Clinical Sciences, Sahlgrenska Academy at University of Gothenburg, Gothenburg, Sweden

**Keywords:** blood glucose, carbohydrates, continuous glucose monitoring, insulin, physical activity, time in range, type 1 diabetes

## Abstract

**Background:** Prolonged physical exercise (PE) is a challenge in type 1 diabetes with an increased incidence of both hypoglycemia and hyperglycemia.

**Purpose:** To evaluate the impact of two consecutive days of carbohydrate (CHO) loading, followed by high intermittent CHO-intake during prolonged PE, facilitated by a proactive use of Real-Time Continuous Glucose Monitoring (rtCGM), on glucose control in individuals with type 1 diabetes.

**Methods:** Ten physically active individuals with type 1 diabetes were invited to participate in a 3-day long sports camp with the objective to evaluate CHO-loading and high intermittent CHO-intake during prolonged PE. 1.5 months later the same procedure was evaluated in relation to a 90 km cross-country skiing race (Vasaloppet). Participants were instructed to act proactively using rtCGM with predictive alerts to maintain sensor glucose values within target range, defined as 72–180 mg/dl (4–10 mmol/l).

**Results:** Mean glucose values during CHO-loading were: day 1; 140.4 ± 45.0 mg/dl (7.8 ± 2.5 mmol/l) and day 2; 120.6 ± 41.4 mg/dl (6.7 ± 2.3 mmol/l). Mean sensor glucose at start of PE was 126.0 ± 25.2 mg/dl (7.0 ± 1.4 mmol/l) and throughout PE 127.8 ± 25.2 mg/dl (7.1 ± 1.4 mmol/l). Percentage of time spent in range (TIR) respective time spent in hypoglycemia was: CHO-loading 74.7/10.4% and during PE 94.3/0.6%.

**Conclusions:** High intermittent CHO-intake during prolonged PE combined with proactive use of rtCGM is associated with good glycemic control during prolonged exercise in individuals with diabetes type 1. However, the time spent in hypoglycemia during the 2-days of CHO-loading was 10.4% and therefore a lower insulin dose might be suggested to reduce the time spent in hypoglycemia.

**Clinical Trial Registration:** www.ClinicalTrials.gov, identifier NCT03722225

## Introduction

Studies have shown that individuals with diabetes type 1 have the same aerobic capacity as healthy individuals, provided that blood glucose (BG) is maintained in the euglycemic range (1, 2). However, in individuals with diabetes type 1, physical exercise (PE) is associated with an increased incidence of both hypoglycemia and hyperglycemia (3, 4). Whether the aim of PE is to have fun, become physically fit, lose weight or to increase the physical performance, the general glucose management strategy during PE is to avoid hypoglycemia. Depending on the specific aim, different strategies are required to obtain stable glucose control which could be achieved via alterations in the carbohydrate (CHO) intake or insulin adjustments or a combination of these.

In healthy subjects, new guidelines are available (5), with information on optimal CHO-intake for PE with various durations in order to ensure maximum performance and achieve stable blood glucose levels (5). A CHO-intake of 90 g/h during prolonged PE (>2.5/h) is recommended to maximize performance. The high CHO-intake contributes to a maintained high rate of CHO oxidation necessary to sustain the exercise intensity and to reach better performance (6).

For individuals with diabetes type 1 there are currently no recommendations concerning insulin adjustments and/or the CHO-intake during prolonged endurance sports when physical performance is to be considered. In the absence of such recommendations, the individuals must adopt a trial-and-error approach based on their past experience of BG responses to similar activities (7, 8).

In healthy individuals an increased intake of carbohydrates (CHO-loading) during the days before prolonged PE (>1.5 h) was shown to improve the physical performance (9). In healthy individuals an increased intake of carbohydrates, such as carbohydrate loading (CHO), is automatically accompanied by an increased insulin secretion with maintenance of a stable glucose level. In individuals with type 1 diabetes, this automatic regulation is missing and to our knowledge, only one study has been published investigating the effect of CHO-loading on physical performance and blood glucose control (10). In this study, the glucose control deteriorated along with impaired physical performance and it was concluded that a high CHO diet prior to PE was not recommended in the case that blood glucose control cannot be maintained in individuals with type 1 diabetes. Given the limited knowledge, it is therefore important to develop effective CHO-loading models for the individual with type 1 diabetes.

A common strategy prior to the prolonged PE in individuals with diabetes type 1 is to reduce the basal insulin doses by about 30–80% to reduce the risk of hypoglycemia. However, during competition, problems with hyperglycemia is often seen and is due to stress-induced release of glucose elevating stress hormones such as adrenaline and noradrenaline (7, 8, 11). A hyperglycemia at this time might result in avoidance of ingesting CHO at the start of the race and, which in turn can result in a late onset hypoglycemia, due to the increased requirements of CHO during the PE have not been met (8).

Furthermore, during prolonged PE, the carbohydrate intake has primarily been governed by the current blood glucose value or trend in previous studies (7, 8) instead of actually adding the amount of CHO required for the duration and intensity of the exercise. This reversed approach was evaluated when individuals with type 1 diabetes conducted a 90 km cross-country skiing race (Vasaloppet) (12). A fixed amount of CHO (75 g/h) was used corresponding to the duration of the PE aiming to maintain high intensity during the activity. In this study, the adjustments of insulin doses were individualized and slightly increased for the majority of participants. This strategy resulted in a maintained good glycemic control during the race.

With technological developments in recent years it is not only possible to accurately assess glucose control but also to take preventive actions. Real-Time Continuous Glucose Monitoring (rtCGM) has been shown to improve glycated hemoglobin (A1c) as well as reducing the risk of hypoglycemia (13). As hypoglycemia and hyperglycemia is associated with PE, rtCGM may therefore improve glucose management related to PE (12). The rtCGM allowed access to combined glucose information such as; actual value, glucose trend and direction and rate of the glucose changes. This information could be used to promote proactive actions, to reduce the glucose variability and if sustained also to reduce the A1c level (13–16).

The aim of this study was to evaluate CHO-loading prior to and intermittent high carbohydrate intake during prolonged PE in individuals with type 1 diabetes and its impact on glycemic control when applied along with rtCGM.

## Materials and Methods

### Study Population

Physically active individuals with type 1 diabetes from different regions of Sweden reported their interest to participate in the study and 10 individuals were selected.

Inclusion criteria: age 18–50 years, type 1 diabetes with a diabetes duration >1 year, exercising regularly ≥3 workout/week, previous experience from long-distance cross-country skiing and willingness to follow the study according to the protocol.

Exclusion criteria: A1c 8.6% NGSP (70 mmol/mol IFCC) proliferative retinopathy, known nephropathy or cardiac failure, and presently following a low-carbohydrate diet.

### Study Design

This is a descriptive, single arm, non-randomized interventional study. The intervention consisted of CHO-loading prior to and intermittent high CHO-intake during PE and a proactive use of rtCGM to achieve and maintain glucose control. A 3-day long sports camp was performed 1.5 months before a 90 km cross-country skiing race (Vasaloppet). The objective of this sport camp was to educate and prepare the participants on CHO-loading and the extended use of CHO during PE. All participants used rtCGM during the sports camp, the time between the sports camp and the Vasaloppet as well as during the race.

### Preparation for Exercise—Glucose Control During 2 Days of Carbohydrate Loading

All participants conducted a 2-day CHO-loading twice, first during the time interval between the sports camp and the Vasaloppet and later during the 2 days prior to the race. The first occasion was used as an exercise in adjusting the insulin doses. The CHO-loading procedure consisted of the usual diet extended by 2 g of CHO/kg/day for 2 days in the form of a sports drink mixed in 1 liter of water which was ingested, 0.4 dl/h every half hour, intermittently for 12 h (08:00 a.m.−08:00 p.m.). The extended CHO-intake was simultaneously balanced with an increased amount of basal insulin where the individual carbohydrate to insulin ratio was used to find the appropriate dose to add as a basal dose during the subsequent 12 h. Furthermore, before bedtime after the start of CHO-loading the basal insulin was increased by ~20% day 1 and 30% during day 2 lasting throughout the night until breakfast on both nights. The participants on multiple daily injection (MDI) therapy administered the long-acting insulin twice daily with a 50–50% distribution morning and evening. During the CHO-loading, the long-acting insulin taken in the evening was increased by 20% prior to night 1 and by 30% prior to night 2. Extra bolus doses were used during daytime corresponding to the added carbohydrate amount. Supplementary File 1 illustrates the CHO-loading procedure including recommended insulin dose adjustments.

### Preparation for Exercise—Glucose Control Between Meal and Start of the Race

To avoid the stress-induced hyperglycemia prior to the start of the Vasaloppet, the participants practiced to deliberately increase the mealtime insulin dose prior to PE during preparations prior to the race aiming to reach a glucose target of 90–144 mg/dl (5–8 mmol/l) at the start of PE. The breakfast was consumed at least 2 h before the start of PE and the participants were given a breakfast they were used to consume in relation to PE. The participants were informed to consume extra CHO (about 20–25 g) in the form of a sport drink, or to use a 30-min pump suspension, if the glucose level tended to decrease toward hypoglycemia before start, thus using the rtCGM proactively.

### Implementation—Glucose Control During the Race

During all exercise sessions, the participants consumed a glucose–fructose containing liquid, three times per hour. The use of this mixed glucose-fructose sports drink enabled the extended use of CHO, corresponding to 1.00 ± 0.15 g CHO/kg body weight. The participants with type 1 diabetes were instructed to balance this CHO-intake with an appropriate insulin dose aiming at glucose values in range 72–180 mg/dl (4–10 mmol/l).

### rtCGM

The Dexcom G5 Platinum system (Dexcom, San Diego, CA) was used during the study period. The sensors were inserted and calibrated according to company recommendation. The rtCGM devices and insulin pumps were downloaded via Diasend (Glooko Inc., Mountain View, CA) downloading system. HemoCue Glucose 201 RT (HemoCue, Ängelholm, Sweden), glucose measuring range 0–180 mg/dl (0–24.6 mmol/l), coefficient of variation (CV) 1.8%, was used for calibration of the rtCGM. All participants utilized the information they received via rtCGM but received no further instructions on glucose management during the race.

Each participant was informed to take proactive actions against hyperglycemia, >180 mg/dl (>10 mmol/l) as well as against hypoglycemia, <72 mg/dl (<4 mmol/l).

Hyperglycemia: Glucose value >180 mg/dl (>10 mmol/l) and stable alternatively increasing trend—Action: Bolus correction aiming at 108 mg/dl (6 mmol/l) taking into account a doubled Insulin Sensitivity Factor (ISF) during exercise.

Hypoglycemia: Glucose value 86.4–102.6 mg/dl (4.8–5.7 mmol/l) + arrow trend obliquely downwards, 102.6–117 mg/dl (5.7–6.5 mmol/l) + single arrow downwards and 117 mg/dl (>6.5 mmol/l) + double arrow downwards—Action: Immediate response by extra CHO-intake (about 20–25 g) or to use a 30-min insulin pump suspension.

### Statistical Analysis

The statistical package for the social sciences (SPSS) version 17.0 (SPSS Inc., Chicago, IL) was used for statistical analysis. Glucose values are presented as mean ± standard deviation unless otherwise indicated.

## Results

Ten individuals, mean age 36.3 ± 4.9 years (range 31–43), mean diabetes duration 15.3 ± 10.9 years (range 1.6–30) and the mean A1c was 7.2% (55 mmol/mol) were included. The baseline characteristics of the study population is shown in Table 1.

**Table 1:** Characteristics of study population.

Number (*n*)	10
Gender (female/male), number	2/8
Age (years), mean ± SD (range)	36.5 ± 4.9 (27–43)
BMI (kg/m^2^), mean ± SD (range)	24.7 ± 2.6 (20.1–27.8)
Weight (lb), mean ± SD (range)	180.6 ± 26 (143.3–233.7)
Weight (kg), mean ± SD (range)	81.9 ± 11.8 (65–106)
Diabetes duration (years), mean ± SD (range)	15.3 ± 10.9 (1.6–30)
Treatment regimen (CSII/MDI), number	7/3
Total daily dose of insulin (IU/kg), mean (range)	0.47 (0.17–0.81)
A1C (NGSP, %), mean (range)	7.2 (6.3–8.3)
A1C (IFCC, mmol/mol), mean (range)	55 (45–67)

*CSII, Continuous Subcutaneous Insulin Infusion; MDI, Multiple Daily Injections; NGSP, Glycohemoglobin Standardization Program; IFCC, International Federation of Clinical Chemistry*.

### Preparation for Exercise—Glucose Control During 2 Days of Carbohydrate Loading

During the CHO-loading prior to the Vasaloppet, one participant had problems with the rtCGM equipment during day 1 resulting in missing data. Mean glucose during the 2-day CHO-loading were: for both days; 129.6 ± 43.2 mg/dl (7.2 ± 2.4 mmol/l), day 1; 140.4 ± 45.0 mg/dl (7.8 ± 2.5 mmol/l) and day 2; 120.6 ± 41.4 mg/dl (6.7 ± 2.3 mmol/l) (Table 2). TIR defined as 72–180 mg/dl (4–10 mmol/l) was: for both days; 74.4%, day 1; 70.7% and day 2; 78.3%. Time spent in hypoglycemia <72 mg/dl (<4 mmol/l) was: for both days; 10.4%, day 1; 9.9% and day 2; 10.8%. The distribution of glucose values in TIR, hyperglycemia and hypoglycemia is shown in Table 3.

**Table 2:** Mean glucose levels measured by rtCGM in individuals with type 1 diabetes during a 2-day carbohydrate loading prior to Vasaloppet.

**Glucose control during two days of carbohydrate loading prior to Vasaloppet**								
**Participants**	**Day 1**				**Day 2**			
	**Glucose control mg/dl (mmol/l)**				**Glucose control mg/dl (mmol/l)**			
	**Mean**	**SD**	**Min**	**Max**	**Mean**	**SD**	**Min**	**Max**
A	153 (8.5)	59 (3.3)	40 (2.2)	277 (15.4)	140 (7.8)	56 (3.1)	40 (2.2)	259 (14.4)
B	126 (7.0)	49 (2.7)	40 (2.2)	275 (15.3)	101 (5.6)	40 (2.2)	40 (2.2)	220 (12.2)
C	113 (6.3)	22 (1.2)	47 (3.6)	173 (9.6)	106 (5.9)	23 (1.3)	52 (2.9)	185 (10.3)
D	155 (8.6)	31 (1.7)	88 (4.9)	232 (12.9)	142 (7.9)	43 (2.4)	70 (3.9)	245 (13.6)
E	95 (5.3)	31 (1.7)	40 (2.2)	194 (10.8)	94 (5.2)	34 (1.9)	41 (2.3)	230 (12.8)
F	162 (9.0)	49 (2.7)	56 (3.1)	286 (15.9)	149 (8.3)	34 (1.9)	68 (3.8)	212 (11.8)
G	180 (10.0)	67 (3.7)	49 (2.7)	297 (16.5)	119 (6.6)	45 (2.5)	54 (3.0)	268 (14.9)
H	NA	NA	NA	NA	146 (8.1)	61 (3.4)	59 (3.3)	238 (13.2)
I	155 (8.6)	36 (2.0)	45 (2.5)	236 (13.1)	119 (6.6)	41 (2.3)	47 (2.6)	218 (12.1)
J	131 (7.3)	67 (3.7)	40 (2.2)	326 (18.1)	117 (6.5)	52 (2.9)	43 (2.4)	265 (14.7)
All	140 (7.8)	45 (2.5)	50 (2.8)	256 (14.2)	121 (6.7)	41 (2.3)	47 (2.6)	234 (13.0)

*In addition to the usual diet, the participants added 2 g of carbohydrates/kg/day*.

**Table 3:** Glucose values in individuals with type 1 diabetes during 2 days of carbohydrate loading and during a 90 km cross-country skiing race (Vasaloppet).

**Distribution of glucose values as percentage of total time during the carbohydrate loading and at start as well as during Vasaloppet**					
	**Two days of carbohydrate loading**			**Vasaloppet**	
	**Day 1**	**Day 2**	**Both days**	**At start of Vasaloppet**	**During Vasaloppet**
Time in range (% of total time): 72–180 mg/dl (4–10 mmol/l)	70.7	78.3	74.7	100	94.3
Time spent in hypoglycaemia (% of total time): (<72 mg/dl) (<4 mmol/l)	9.9	10.8	10.4	0	0.6
Time spent in hyperglycemia (% of total time): (>180 mg/dl) (>10 mmol/l)	19.4	10.9	14.9	0	5.2

*Time in glucose target range (TIR) (%), defined as 72–180 mg/dl (4–10 mmol/l)*.

### Glucose Control Before and During the Race

The mean insulin bolus dose before the race was increased by 55.5%, from the calculated dose of 5.4 ± 3.0 IU to 8.4 ± 4.0 IU. Mean sensor glucose levels at the start (07:00 a.m.) of the race was 126.0 ± 25 mg/dl (7.0 ± 1.4 mmol/l), with a range of 81–157 mg/dl (4.5–8.7 mmol/l) (Table 4). Thus, 100% of the sensor glucose values were within TIR prior to the race (Table 3).

**Table 4:** Mean glucose levels, carbohydrate intake, and adjustment of basal insulin in individuals with type 1 diabetes during a 90 km cross-country skiing race (Vasaloppet) and the duration of the physical activity (A–J).

**Glucose control and finishing time during a 90 km intensive cross-country skiing race**								
**Participants**	**Treat-ment (MDI/CSII)**	**Glucose value at start mg/dl (mmol/l)**	**Glucose control during Vasaloppet**			**CHO intake/h of activity (g)**	**Basal insulin percentage change (%)**	**Time from start to finish (hour:minutes)**
			**Mean**	**SD**	**Range**			
A	MDI	94 (5.2)	86 (4.8)	8 (0.5)	74–99 (4.1–5.5)	90	0	05:33
B	MDI	140 (7.8)	138 (7.7)	9 (0.5)	119–158 (6.6–8.8)	75	0	05:21
C	CSII	104 (5.8)	129 (7.2)	33 (1.8)	86–194 (4.8–10.8)	75	0	07:00
D	MDI	157 (8.7)	110 (6.1)	22 (1.2)	77–196 (4.3–10.9)	75	0	08:23
E	CSII	153 (8.5)	138 (7.6)	18 (1.0)	99–162 (5.5–9.0)	84	0	05:54
F	CSII	119 (6.6)	150 (8.3)	26 (1.5)	104–184 (5.8–10.2)	84	0	06:45
G	CSII	81 (4.5)	141 (7.8)	31 (1.7)	81–187 (4.5–10.4)	100	0	06:24
H	CSII	142 (7.9)	132 (7.3)	39 (2.2)	68–203 (3.8–11.3)	75	0	07:20
I	CSII	128 (7.1)	128 (7.1)	21 (1.2)	85–158 (4.7–8.8)	75	+27	06:02
J	CSII	139 (7.7)	126 (7.0)	38 (2.1)	83–220 (4.6–12.2)	75	Start: 0 ^#^01:00: −14 ^#^05:30: −31	06:07
All		126 (7.0)	128 (7.1)	25 (1.4)	68-220 (3.8–12.2)	80.8		06:28

# *The participant J had to deviate from the planned CHO intake during the race due to gastrointestinal discomfort which resulted in a reduction of the basal insulin dose 01:00 respectively 5:30 h after start*.

Mean sensor glucose during the race was 127.8 ± 25.2 mg/dl (7.1 ± 1.4 mmol/l) (Table 4). The percentage of TIR was 94.3%, and time spent in hypoglycemia; 0.6% (Table 3).

**Figure 1:**
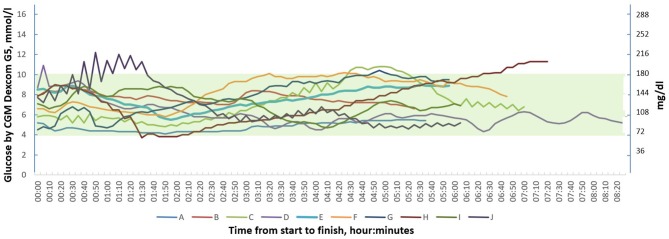
Continuous glucose monitoring graphs of 10 individuals with type 1 diabetes during the 90 km cross-country skiing race (Vasaloppet).

Five of the participants experienced hyperglycemia during the race with a max sensor glucose value 220 mg/dl (12.2 mmol/l) and one subject had a hypoglycemia with a nadir sensor glucose value of 68 mg/dl (3.8 mmol/l). Each participant's glucose graph is shown in Figure 1.

The time needed to complete the race, CHO-intake and basal insulin dose adjustments are illustrated in Table 4.

## Discussion

In this study we have shown that it was possible to achieve and maintain good glycemic control, even during extraordinary challenges such as 2 days of CHO-loading followed by high intermittent CHO-intake during a 90 km long cross-country skiing race. A proactive use of rtCGM enabled individual insulin dose adjustments under these conditions.

To our knowledge few studies have been published regarding models for carbohydrate loading in type 1 diabetes individuals and the same also applies for the use of high carbohydrate intake during prolonged physical exercise. At the same time, we want to emphasize that attempts have been made exploring this area, for example via projects as “Team Novo Nordisk Pro Cycling,” but published data are limited or missing.

### Preparation for Exercise—Glucose Control During 2 Days of Carbohydrate Loading

In the study by McKewen et al. (10), who investigated the effect of increased CHO-intake and its effects on exercise performance and glycemic control in individuals with type 1 diabetes, the participants increased the CHO-intake by 10%, and the total daily dose of insulin by 14%. Despite this, the mean blood glucose increased 10% compared to normal diet 171 ± 25.2 mg/dl (9.5 ± 1.4 mmol/l) vs. 154.8 ± 30.6 mg/dl (8.6 ± 1.7 mmol/l), *p* = 0.005. In contrast to this we obtained preserved glucose control during a 2-day carbohydrate loading. An explanation to retained glucose control in our study may be due to the use of gradually increased levels of basal insulin during night 1 (+20%) and night 2 (+30%). Studies have suggested that increased muscular glycogen content increases insulin resistance which could explain why the participants required more insulin during night 2 (17–19). It might be suggested that a slightly lower basal insulin rate could be recommended in the present study considering that 10.4% of the time was spent in the hypoglycemic range. It should also be considered that individuals with type 1 diabetes usually face problems with hypoglycaemia on regular basis. This will affect the counterregulatory hormonal response and attenuate endogenous hepatic glucose production during exercise and might therefore result in an increased risk of hypoglycaemia during a subsequent physical activity (20). Thus, avoidance of a hypoglycemia the day before prolonged physical activity is most likely even more important with the approach presented in this study where the basal doses were not reduced before and during the race.

### Preparation for Exercise—Glucose Control Between Meal and Start of the Vasaloppet

Prior to competitions, stress is common, causing increased release of adrenaline and noradrenaline (21). This stress results in hyperglycemia (7, 8, 11). Beyond this fact, individuals with type 1 diabetes often have a fear of hypoglycemia associated with PE, which often results in a reduction of the basal insulin dose before the start of the race (11). In a recent publication Riddell et al. showed that the variability of glucose control during aerobic exercise partially was explained by pre-exercise glucose level (22). Our approach was to reach a stable and good glucose control prior to the start of the race. To reduce the risk of hyperglycemia in this study, first the participants did not have to reduce basal insulin before and during the competition due to the plan we had about using an extended amount of CHO throughout the race. Second, the participants increased the insulin dose to the meal taken 2.5 h before the start by mean 55.5%. To our knowledge there are no previous studies using this novel model. The rtCGM, used in our study, provided safety to this approach as the individual could be warned about pending hypoglycemia and consume a liquid CHO solution in such occasions.

### Implementation—Glucose Control During the Race

Both hypoglycemia and hyperglycemia are important to avoid during PE when performance also is regarded as an important factor. Hyperglycemia increases the release of free fatty acids (FFA) (23). An increase in FFA has been shown to decrease both insulin-dependent and insulin-independent muscular glucose uptake (24–26). In one of these studies an increase in plasma FFA up to 31.1 mg/dl (1.1 mmol/l) was shown to decrease muscle glucose uptake by 42% (25). In addition, an increase in plasma FFA will reduce muscle CHO oxidation, which may adversely affect physical performance (27).

Despite the prolonged duration of the PE in the current study, the participants spent 94.3% of the TIR. Our results differ in comparison to a previous study in individuals with type 1 diabetes who performed a 75 km cross-country skiing race (>7 h) at two consecutive years (1986 and 1987), where a CHO-intake corresponding to 40 g of CHO/h appeared to prevent hypoglycemia in the majority of the participants (8 of 9) in both competitions (7). In the same study, mean plasma free insulin levels in the individuals with type 1 diabetes before the race were half (7.8 ± 2.0 mU/l) of that was seen in healthy control subjects (15.6 ± 3.1 mU/1, *p* < 0.001) while mean pre-race blood glucose for the participants with type 1 diabetes was 365.4 ± 32.4 mg/dl (20.3 ± 1.8 mmol/l). Thus, compared to this study, we were able to achieve a better glycemic control at start and then also to maintain this glucose control during prolonged PE.

A possible mechanism behind the good results in our study could be the combination of carbohydrate loading and subsequent high carbohydrate intake during prolonged physical exercise. This procedure ensured a relatively high glycogen content in the liver and muscles prior to exercise. Exercise performance/capacity and a stable plasma glucose level is often limited by endogenous carbohydrate availability during prolonged physical activity (28). In the current study, the participants consumed between 75 and 100 g CHO/h during the race. The main focus of this study was to continuously ingest increased amounts of carbohydrates to maintain a high carbohydrate availability throughout the race and thereby hopefully achieve a preserved blood glucose value. However, it should be highlighted that an increased intake of carbohydrates during exercise does not seem to spare muscle glycogen (29, 30). Instead, studies have shown that an increased carbohydrate intake spare the liver glycogen which likely supports both a stable blood glucose and performance in the latter stages of prolonged exercise (31, 32).

Gastrointestinal discomfort is very common symptom during exercise, especially in prolonged endurance races (33). The occurrence of gastrointestinal disturbances has been related to the CHO-intake during exercise (34). However, it has been shown that the gut is adaptable in that the intestinal capacity to absorb CHO can be increased by regularly consuming increasing amounts of CHO during exercise (35, 36). In the current study, the participants had a high intake of CHO corresponding to 75–100 g/h during the race. Unfortunately, one participant had to deviate from the planned CHO-intake during the race due to gastrointestinal discomfort. This deviation resulted in a reduction of the planned basal insulin dose. This participant had, due to an upper respiratory infection, 2 weeks prior to the Vasaloppet, only completed a few exercise sessions with a higher intake of CHO before the PE. It is likely that this participant would have needed more exercise sessions with a high CHO-intake to increase the tolerance for high amounts of CHO.

### Limitations

This study was performed as an exploratory study which includes the benefits of this being a real world situation. As opposed to this, there are of course also limitations as a control group is missing and where the environment made it difficult to carry out parallel sampling to evaluate the mechanisms behind the good glucose control achieved in the study.

Furthermore, the participants was not randomly selected and could thus limit the generalisability of the study. Vasaloppet is a 90 km long cross-country skiing race which is very demanding in terms of individual physical performance and the participants had to have relative high level of fitness to be able to complete the race. Therefore, the results could be seen as a description of real-world data in this specific group.

## Conclusions

We conclude, that high intermittent CHO-intake during prolonged PE was associated with good glucose control in individuals with type 1 diabetes. However, the proportion of time spent in hypoglycemia during the 2-days of CHO-loading was 10.4% and a lower insulin dose might have been required to reduce time spent in hypoglycemia. rtCGM could be beneficial when used proactively to maintain sensor glucose values within target range before and during PE. These strategies and the mechanisms that create the conditions for good glucose control during prolonged physical exercise needs to be further evaluated in randomized controlled studies.

## Ethics Statement

All procedures performed in this study involving human participants were in accordance with the ethical standards of the national research committee and with the 1964 Helsinki declaration and its later amendments. Signed informed consent was collected from all participants prior to study start. This study was approved by the Regional Ethical Review Board in Uppsala, Sweden (DNR: 2012/159).

## Author Contributions

SM and PA conceived and designed research, conducted the experiments, and analyzed data. JJ participated in the planning of the study. SM, PA, and JJ did all participate during the preparatory sports camp. SM wrote the manuscript. PA and JJ reviewed the manuscript. All authors read and approved the manuscript.

### Conflict of Interest Statement

The authors declare that the research was conducted in the absence of any commercial or financial relationships that could be construed as a potential conflict of interest.

## Abbreviations

A1c: Glycated Hemoglobin
BG: Blood Glucose
BMI: Body Mass Index
CHO: Carbohydrates
CSII: Continuous Subcutaneous Insulin Infusion
IFCC: International Federation of Clinical Chemistry
MDI: Multiple Daily Injections
NGSP: National Glycohemoglobin Standardization Program
PE: Physical Exercise
rtCGM: Real-Time Continuous Glucose Monitoring
TIR: Time in range.
