# Supplementary material for: Carbohydrate Loading Followed by High Carbohydrate Intake During Prolonged Physical Exercise and Its Impact on Glucose Control in Individuals With Diabetes Type 1—An Exploratory Study
Source: Front Endocrinol (Lausanne). 2019 Aug 21;10:571. doi: 10.3389/fendo.2019.00571 (PMC6712943; doi:10.3389/fendo.2019.00571)
Supplement: Supplementary file 1 [file Data_Sheet_1.PDF]

Performing a 2-day carbohydrate loading

When carry out a 2-day carbohydrate loading, the increased carbohydrate intake will lead to an increased need for insulin.

More insulin is partly needed for the extra carbohydrates but also later during the night as a basal/long-acting insulin.

An increased content of muscle- and liver glycogen content tends to be the reason for this.

Extra carbohydrates first step

During the carbohydrate loading:

- continue to eat as usual
- add a carbohydrate-containing fluid, to be consumed during 12 hours, 8am to 8pm
- add 2 grams of carbohydrates **x** your body weight as this fluid, e.g. 50 kg bodyweight x 2 = 100 g extra carbohydrates day 1 and 2, mixed in 1 litre of water

Extra insulin first step

The "500 rule" is a way to estimate how many grams of carbohydrate that will be covered by one unit of insulin. This is your "insulin to carb ratio" or your "carb factor".

- calculate 500/TDD (Total Daily Dose of insulin) = X grams of carb covered by one unit of insulin, e.g. 500/50 =10, which means 10 g of carbohydrates is covered by 1 unit of insulin

Important information

There are always individual differences.

Try to carry out one carbohydrate loading at home.

Use our recommendation as a starting point and adjust step by step depending on results.

Stig Mattsson, Dietician, PhD student, Institute of Medical Sciences, Örebro University, Örebro, Sweden
Peter Adolfsson, MD, PhD, Institute of Clinical Sciences, Sahlgrenska Academy at University of Gothenburg, Gothenburg, Sweden

Extra insulin covering the extra carbohydrates -– day 1

- if you intend to drink e.g. 100 g extra carbohydrates, in addition to other meals, and your “carb factor” is 10. This means that the basal insulin dose should be increased by 10 units (100g/10 = 10 units).
- in practical terms (our experience) then reduce this amount of insulin by about 15%, e.g. 10 Units x 0.85 = 8.5 U
- mix the 100 g extra carbohydrate with 1 litre of water to a concentration of 10 g carbohydrates per 100 ml
- this amount is then consumed during 12 hrs which means 1000 ml/ 12 hrs = 80 ml per hour or 40 ml per 30 min during these 12 hours
- in case of CSII: increase the basal insulin dose by in total 8.5 U, evenly distributed during the 12 hrs = 0.70 U extra per hour to be added to the normal basal dose per hour
- in case of MDI: add extra fast-acting insulin evenly distributed during the 12 hours as small doses every 2-3 hours, starting at the start time for extra carbohydrates

Insulin dose to meals

- use your normal insulin-carbohydrate ratio for meals day 1

Insulin correction factor

- use your normal insulin sensitivity factor/correction factor during day 1

Basal insulin dose during night – day 1 (after 20.00 to breakfast next morning)

- after 20.00 day 1, increase the basal insulin dose overnight until breakfast the next morning by about 20%. This recommendation is based on the experience we learned during the Vasaloppet 2014 and Vasaloppet 2015
- e.g. if the normal basal dose is 1 u/hr, it means increase this to 1.2 U/h during the night

Stig Mattsson, Dietician, PhD student, Institute of Medical Sciences, Örebro University, Örebro, Sweden
Peter Adolfsson, MD, PhD, Institute of Clinical Sciences, Sahlgrenska Academy at University of Gothenburg, Gothenburg, Sweden

Extra insulin covering the extra carbohydrates -– day 2

- in our example during day 1, we increased the basal insulin by + 0.70 IU/h to cover for the extra carbohydrates given as liquid
- from previous tests we have learned that it may be appropriate to increase this amount of insulin by about 20% during day 2, meaning 0.70 x 1.2 = 0.85
- the 0.85 U/h is then added extra per hour to be added to the normal basal dose per hour
- again the 100 g extra carbohydrates is mixed with 1 litre of water to a concentration of 10 g carbohydrates per 100 ml
- this amount is then consumed during 12 hrs which means 1000 ml/ 12 hrs = 80 ml per hour or 40 ml per 30 min during these 12 hours
- in case of CSII: increase the basal insulin dose by 0.85 U extra per hour to be added to the normal basal dose per hour
- in case of MDI: add extra fast-acting insulin evenly distributed during the 12 hours as small doses every 2-3 hours, starting at the start time for extra carbohydrates

Insulin dose to meals

- use your normal insulin-carbohydrate ratio for meals day 1

Insulin correction factor

- use your normal insulin sensitivity factor/correction factor during day 1

Basal insulin dose during night – day 2 (after 20.00 to breakfast next morning)

- during night 2, you will need to increase the basal insulin dose by about 30% compared to regular dose. This recommendation is based on the experience we learned during the Vasaloppet 2014 and Vasaloppet 2015.

Stig Mattsson, Dietician, PhD student, Institute of Medical Sciences, Örebro University, Örebro, Sweden
Peter Adolfsson, MD, PhD, Institute of Clinical Sciences, Sahlgrenska Academy at University of Gothenburg, Gothenburg, Sweden
